# Annual Oration Delivered before the Philadelphia Medical Society, by Appointment, at the Opening of Its Session of 1844-5

**Published:** 1845-03

**Authors:** 


*Annual Oration delivered before the Philadelphia Medical Society, by appointment, at the opening of its Session of* 1844–5. By D. FRANCIS CONDIE, M. D., Honorary Member of the Society. Published by order of the Society.

The theme of this discourse is the "Condition and future prospects of the Medical Profession in the United States." A few extracts will serve to show the general tenor and style of the Address.

"There is a strange fastidiousness exhibited by the leading members of the medical profession, in relation to the discussion of those means by which its interests, as a body, may be promoted, and its legitimate influence with the public secured. They are willing that its success should rest solely upon the character and conduct of its members, and that all extraneous means of support and protection should be rejected; in the full confidence, that the important benefits which the practitioner of the healing art, by the exercise of his skill, confers upon society—and the elevation of mind, and dignity of character, which should distinguish him as a member of a liberal, learned, and highly disinterested profession, will, of themselves, be always sufficient to command the respect, esteem, and confidence of every class of the community, and to secure all the power and influence which physicians as a body, have any right to claim or to expect.

However honourable such sentiments are to those who entertain and act up to them—however much we may desire the general recognition of their truth,—we nevertheless fear, that, in the present state of society, the attempt to carry them out in practice will fail to produce the desired results. We fear, that however legitimate the claim founded merely upon services rendered, or upon superior qualifications alone, it will not be so easily or invariably recognized, nor so readily admitted, as the high-minded members of our profession would fain believe. We have unfortunately to deal, not with what should be, but with what actually exists—without the power of commanding or controlling circumstances—and hence results the necessity of our resorting to *every* honourable means, that is calculated to maintain and further the true interests of the profession, and to erect a barrier, that all may recognize, for the separation of the qualified from the unqualified practitioner—the physician from the pretender—and thus prevent the ultimate degradation and destruction of the medical profession, from allowing its titles, its honours, and its emoluments to be usurped by the host of empirics who now, on every side, encompass it."

In the justness of the following remarks, we apprehend all who read them will concur.

"The true value of the physician—his competency to fulfil the important duties required of him—and his faithfulness in their fulfilment, can, with difficulty, be correctly and fully estimated by any other than a member of his own profession. Medicine is, unquestionably, one of the most difficult sciences properly to acquire—demanding not only the highest grade of natural ability, but long years of close and laborious study, and an acquaintance with many collateral branches of knowledge. It has, indeed, been correctly remarked, that there is scarcely any circle of human learning, upon the boundaries of which the scientific physician does not necessarily infringe, in some point or other of his extensive orbit. In fact, to acquire that extent of information, and that degree of practical skill necessary to constitute the physician, requires the close and devoted application of all the mind's best energies—under circumstances the most favourable to their full occupation and development. And yet almost every one in the community—whether learned or unlearned—assumes to himself the right to decide, not merely upon the competency of the individual members of the medical profession—which, within certain limits, is his right—but upon the relative value of different medical doctrines and modes of practice.

The skill of a physician, and consequently the propriety of the remedial measures pursued by him may, unquestionably, be very fairly tested by the general result of his practice—but to do this requires opportunities for observation, and an accuracy of judgment which the members of the community at large do not possess—their decision is consequently founded, in most instances, upon the result of single cases, the circumstances of which are often but imperfectly understood, or entirely misrepresented.

It is this want of ability in the public to decide correctly upon the value of remedial agents and the competency of medical practitioners, combined with that desire which all alike experience for speedy and certain relief, that renders it, more perhaps than any other cause, the ready dupe of bold pretension and unprincipled empiricism. The vaunted infallibility of any nostrum that promises an immediate restoration of health, becomes far more attractive than the slow and cautious measures pursued by the scientific physician. It is not, therefore, surprising, that the preposterous absurdities of homœopathy—the equally ridiculous and mischievous practices of the Thompsonians, Botanists, and Hydropathists, or the more novel folly of the Crono-thermists, should become more popular than the curative skill exercised by the highest order of medical abilities, accompanied by the greatest natural and acquired advantages."

Dr. Condie enters at considerable length, in the remaining portion of his address, into the consideration of the evils which arise to the profession and the community from the ultra democratical operation of our institutions, and deplores, as all intelligent men must, who take a full view of the matter, the incompetency of the people at large to judge of the qualifications of a physician, and the want of adequate protection from the impositions of ignorance and knavery, so generally practised under the cloak of the healing art. Unfortunately, we have not discovered in the present address any tangible scheme, or plausible suggestion, for abatement of the evil. The liberty of doing as they please, even to the destruction of property, health, and life itself, seems to be vouchsafed to all who rally beneath the glorious "star spangled banner." The right of individual judgment, in any thing but party politics, is not likely soon to be surrendered by our sturdy republican citizens; and physicians, as well as others, must be content to take the evils as well as to enjoy the advantages of our liberal laws and principles. We long thought as our author does on this subject, and toiled shoulder to shoulder with him in the cause of reform, but we have come at last to believe that there is no remedy. We even think that so long as the present system of instituting Colleges for conferring degrees prevails, (and nothing can possibly stop it,) it is well that we have no laws restraining the practice of medicine. The very fact that there are none, is some admonition to the prudent.

---

*Royal College of Physicians of London.*—The following gentlemen passed this college, as extra-licentiates, on the last board day, 26th of December, 1844:—Drs. John Forbes, W. B. McEgan, Septimus Wray, R. I. Scott.—*Lancet.*

